# Outcomes of Induction of Labour in Women with Previous Caesarean Delivery: A Retrospective Cohort Study Using a Population Database

**DOI:** 10.1371/journal.pone.0060404

**Published:** 2013-04-02

**Authors:** Sarah J. Stock, Evelyn Ferguson, Andrew Duffy, Ian Ford, James Chalmers, Jane E. Norman

**Affiliations:** 1 MRC Centre for Reproductive Health, University of Edinburgh, Edinburgh, United Kingdom; 2 NHS Lanarkshire Department of Obstetrics and Gynaecology, Wishaw General Hospital, Wishaw, United Kingdom; 3 Information Services Division, NHS National Services Scotland, Edinburgh, United Kingdom; 4 Robertson Centre for Biostatistics, University of Glasgow, Glasgow, United Kingdom; The University of Adelaide, Australia

## Abstract

**Background:**

There is evidence that induction of labour (IOL) around term reduces perinatal mortality and caesarean delivery rates when compared to expectant management of pregnancy (allowing the pregnancy to continue to await spontaneous labour or definitive indication for delivery). However, it is not clear whether IOL in women with a previous caesarean section confers the same benefits. The aim of this study was to describe outcomes of IOL at 39–41 weeks in women with one previous caesarean delivery and to compare outcomes of IOL or planned caesarean delivery to those of expectant management.

**Methods and Findings:**

We performed a population-based retrospective cohort study of singleton births greater than 39 weeks gestation, in women with one previous caesarean delivery, in Scotland, UK 1981–2007 (n = 46,176). Outcomes included mode of delivery, perinatal mortality, neonatal unit admission, postpartum hemorrhage and uterine rupture. 40.1% (2,969/7,401) of women who underwent IOL 39–41 weeks were ultimately delivered by caesarean. When compared to expectant management IOL was associated with lower odds of caesarean delivery (adjusted odds ratio [AOR] after IOL at 39 weeks of 0.81 [95% CI 0.71–0.91]). There was no significant effect on the odds of perinatal mortality but greater odds of neonatal unit admission (AOR after IOL at 39 weeks of 1.29 [95% CI 1.08–1.55]). In contrast, when compared with expectant management, elective repeat caesarean delivery was associated with lower perinatal mortality (AOR after planned caesarean at 39 weeks of 0.23 [95% CI 0.07–0.75]) and, depending on gestation, the same or lower neonatal unit admission (AOR after planned caesarean at 39 weeks of 0.98 [0.90–1.07] at 40 weeks of 1.08 [0.94–1.23] and at 41 weeks of 0.77 [0.60–1.00]).

**Conclusions:**

A more liberal policy of IOL in women with previous caesarean delivery may reduce repeat caesarean delivery, but increases the risks of neonatal complications.

## Introduction

Rates of caesarean section are increasing worldwide, with rates of more than 32% in the USA [1]. Many women therefore embark on pregnancy with a previous caesarean scar, and the optimal delivery method in this scenario is uncertain. It is well documented that the risks of caesarean section for women increase with increasing numbers of caesarean deliveries. These include potentially life-threatening complications including haemorrhage, surgical complications and morbidly adherent placenta, [2], [3]. Promoting vaginal birth after previous caesarean (VBAC) may help avoid these complications in future pregnancy, but there are risks, particularly for babies. A recent carefully designed prospective restricted cohort study suggested that, when compared to elective repeat caesarean section, attempting VBAC resulted in a significantly greater risk of a composite measure of serious morbidity and death for infants [4]. However, as elective repeat caesarean delivery is usually performed before term, the women having elective repeat caesarean section in Crowther et al's study delivered at a significantly earlier gestation than women attempting VBAC (mean +/− SD 38.8+/−0.7 weeks gestation versus 40.0 +/−1.1 weeks gestation). It is possible that the observed differences in neonatal complications arose because of differences in gestational age at birth, rather than the intended mode of delivery, given that perinatal mortality and infant morbidity increase with advanced gestation beyond term [5]–[7].

As we and others have shown, there is increasing evidence that, in women without a previous caesarean delivery, expediting delivery around term by means of induction of labour (IOL), results in lower perinatal mortality and caesarean delivery rates compared to the alternative of expectant management (allowing the pregnancy to continue to await spontaneous labour or definitive indication for delivery) [8], [9]. However, there are particular concerns about induction of labour in women with a previous caesarean section, with previous influential studies highlighting the risks of uterine rupture, a catastrophic event for both mother and infant [6], [10]. Thus there is uncertainty whether induction of labour to expedite delivery around term in women with a previous caesarean section confers the same benefits as in women without a caesarean scar. The balance between the possible adverse consequences of induction of labour in women with a previous scar, versus the increasing risk of pregnancy complications as gestation advances past term with expectant management [5], [6] and the short and long-term complications associated with routine repeat caesarean delivery [11], [12] is unclear. The lack of robust evidence and the need for further research in this area is highlighted in national professional guidelines relating to IOL and vaginal birth after caesarean delivery [13]–[15]. The objective of this study was to use a population database to describe outcomes of IOL around term (39–41 weeks gestation) in women with one previous caesarean delivery. Furthermore, we aimed to compare the mode of delivery in women with a previous caesarean section undergoing IOL at 39,40 and 41 weeks with those expectantly managed; and to compare rates of neonatal and maternal complications in women in whom delivery is expedited by IOL or elective repeat caesarean delivery at 39, 40 and 41 weeks, both in comparison to expectant management.

## Methods

### Databases

We used the SMR02/SMR11/SBR/SSBIDS/GROS Database, which contains linked maternity, neonatal, and stillbirth/infant death records. The Scottish Morbidity Record 02 (SMR02) records information regarding all women discharged from Scottish maternity units. The level of completeness over the period studied is estimated to be in excess of 98%[16]. SMR11, now replaced by the Scottish Birth Record (SBR) contains information relating to neonatal outcomes. The Scottish Stillbirth and Infant Death Survey (SSBID) contains data on stillbirths and infant deaths that are registered with National Records for Scotland (NRS, previously called the General Register Office for Scotland or GROS), with registration mandated by law after a perinatal death. The linkage is performed using probability matching [17]. All records on the maternity and neonatal file are linked via the mother record. Each year's maternity (SMR02) records are progressively linked to the existing SMR02 records on the database. This provides a file with each mother's maternity records grouped together. Neonatal records, stillbirth and infant death records are then linked to the SMR02 records, which provides the baby information for each pregnancy in the group. Standard International Classification of Diseases (ICD) codes and definitions were used (ICD 9/10). All codes and database fields used are detailed in Table S1.

At commencement of the study, data in the linked maternity database was complete and validated between January1981 and December 2007 – all such deliveries formed our study population.

The Privacy Advisory Committee of the Information Services Division of the National Health Service Scotland provided permission for the record linkage.

### Study Design and Participants

The study is a population-based retrospective cohort study of singleton births at 39 or more weeks gestation, born to women in Scotland, UK, who had one previous caesarean delivery and no previous vaginal deliveries, between 1st January 1981 and 31st December 2007.

### Inclusion/exclusion

We aimed to include women with a live baby who had one previous caesarean delivery, no previous vaginal deliveries, and no recognised contraindications to IOL thus we excluded women with fetal malpresentation, abdominal pregnancy or placenta praevia. We also excluded women who had an antepartum stillbirth from the induction of labour and elective caesarean section groups, if the stillbirth occurred in the same gestational week as delivery. However, as antepartum stillbirth can complicate expectant management, antepartum stillbirths were included in the expectant management comparator group. In Scotland women at term are routinely seen at weekly intervals for antenatal care, which includes auscultation of the fetal heartbeat, and the standard management of antepartum stillbirth is immediate induction of labour. We therefore assumed that all babies in the expectant management group would be alive at the time that induction was initiated in the induction group. Previous studies have used a similar approach [9], [18] supported by analysis of the database that has shown birthweights are not indicative of prolonged maceration. Women with an intrapartum death were included in both groups.

### Comparison groups

The IOL group consisted of women who had IOL at 39 weeks, 40 weeks and 41 weeks gestation, and the elective caesarean section group included women who had a prelabour non-emergency caesarean section at 39 weeks, 40 weeks and 41 weeks gestation. A comparison group was identified representing women who were expectantly managed, and who delivered after the gestation at which IOL or caesarean section was performed. Thus, outcomes of women who underwent IOL or caesarean section and delivered at 39 weeks gestation were compared with outcomes of women who delivered at 40 weeks and beyond; women who underwent IOL or caesarean section and delivered at 40 weeks gestation were compared with women delivered at 41 weeks and beyond; and so on. These comparison groups were chosen as they were felt to best represent the choice available to woman and their caregivers when considering options for delivery. The use of an expectant management group has been advocated as appropriate in a systematic review of studies of IOL [19].

### Outcomes

Mode of delivery was defined as spontaneous vertex delivery, caesarean delivery or operative vaginal delivery (forceps or ventouse). The following complications were recorded (maternal) postpartum haemorrhage and uterine rupture (neonatal) admission of neonate to neonatal or special care baby unit (SCBU) and extended perinatal mortality (defined as stillbirth and death in the first month of life, excluding deaths from congenital anomalies).

### Confounding Factors

The following variables were considered to be potential influences on outcomes, and were included in multiple logistic regression analysis: age group at delivery (categorised as <20, 20–24, 25–29, 30–34, 35–39, 40+ years), period of birth (years 1981–1985, 1986–1990, 1991–1995, 1996–2000, 2001–2007) deprivation quintile (defined by Carstairs 2001 deprivation quintiles 1–5 by postcode[20]) medical or obstetric condition (which included hypertensive or renal disorders, thromboembolic disease, diabetes mellitus, liver disorders, pre-existing medical disorder, antenatal investigation abnormality, suspected fetal abnormality or fetal compromise) and birthweight (categorised as <2500 g; 2500–2999 g; 3000–3499 g; 3500–3999 g; 4000–4999 g; ≥4500 g). The method of IOL (artificial rupture of membranes [ARM], ARM and oxytocin, prostaglandins +/−ARM, prostaglandins and oxytocin +/− ARM) was explored in relation to uterine rupture. The categorisation of adjustment variables was pre-specified before analysis, with the exception of birth weight. In preliminary analyses birth weight was entered into the adjustment model as a continuous variable, as well as categorised at 500 g intervals. Results of both analyses were similar and so categorisation was used. Data regarding body mass index was only collected from 2004 onwards and had a significant number of missing fields, so was not included. Reconfigurations in maternity services during the study period made it unfeasible to adjust for the clustering of women within obstetric unit.

### Statistical Analysis

Univariate analysis was carried out to examine the contribution of each confounding factor in relation to outcomes. Thereafter, we used multivariable logistic regression modelling to examine the relationship between outcomes of IOL, elective caesareans section and expectant management. Missing covariate values were not included in the analysis model. No formal tests of interaction were performed. We included the following confounding factors in the model for mode of delivery: age at delivery, medical complication, birthweight, year of birth and deprivation category. For the models for postpartum haemorrhage, infant mortality and admission to SCBU we also included the mode of delivery. After categorisation, all outcomes were considered as dichotomous variables. The p values for hypothesis tests were two-sided, and the significance level was set at p<0·05, with results presented as odds ratios with 95% confidence intervals (95%CI) unless otherwise stated. Analysis was performed with SPSS version 17.

## Results

**Figure 1:**
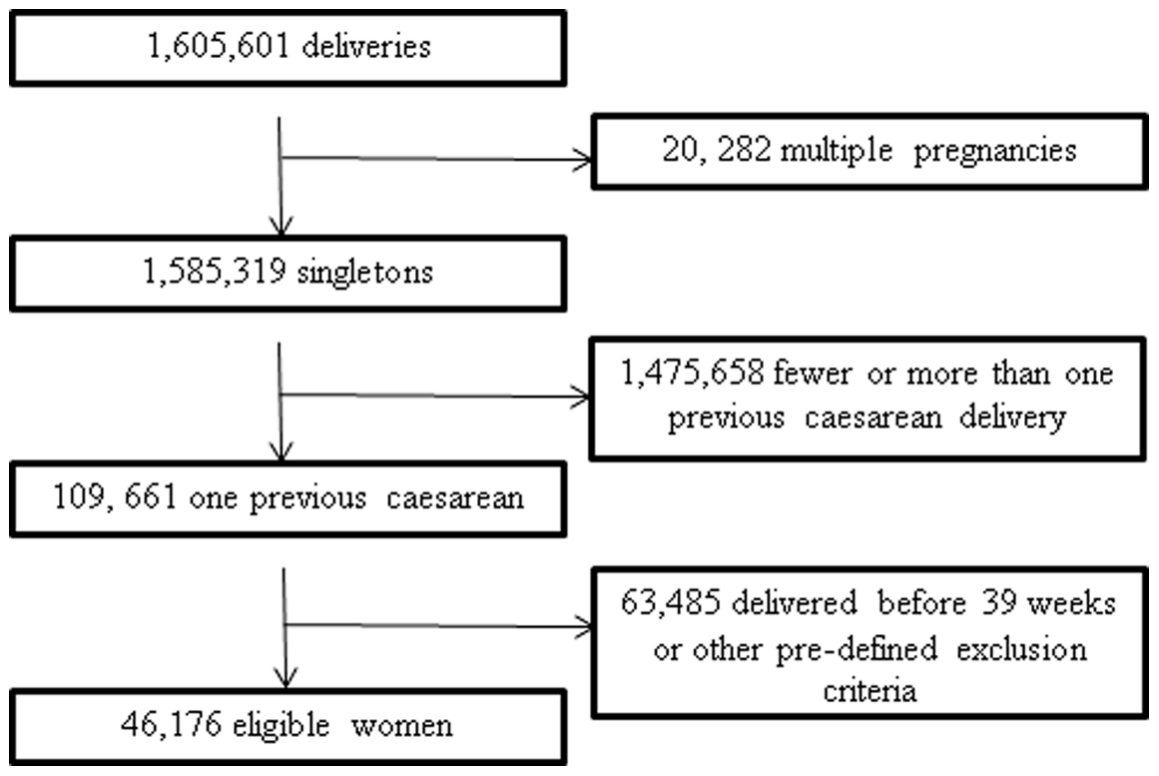
Structure of the cohort. Flow chart showing the structure of the cohort.

Figure 1 summarizes the structure of the cohort. 1,605,601 deliveries were recorded on the database over the study period, including 1,585,319 singleton pregnancies of whom 109, 661 had one previous caesarean delivery and no other deliveries. We excluded 63,485 of these women due to delivery before 39 completed weeks or other predefined exclusion criteria, leaving a cohort of 46,176 women whose only other previous delivery was by caesarean section. Of these, 7,401 underwent IOL, 13,376 had elective repeat caesarean delivery and 25,399 laboured spontaneously.

The characteristics of the cohort are given in table 1.

**Table 1:** Demographics of cohort.

	n (%)
**Age Group**	
<20	946 (2.05)
20–24	8,059 (17.45)
25–29	14,781 (32.01)
30–34	15,190 (32.90)
35–39	6,291 (13.62)
40+	909 (1.97)
**Birthweight Group (g)**	
<2500	1,116 (2.42)
2500–2999	6,377 (13.81)
3000–3499	16,750 (36.27)
3500–3999	15,209 (32.94)
4000–4499	5,537 (11.99)
4500+	1,163 (2.52)
Missing	24 (0.05)
**Year of birth**	
1981–1985	7,216 (15.63)
1986–1990	7,947 (17.21)
1991–1995	8,972 (19.43)
1996–2000	8,664 (18.76)
2001–2007	13,377 (28.97)
**Carstairs 2001 deprivation quintiles**	
1 (least deprived)	9,168 (19.85)
2	9,153 (19.82)
3	8,671 (18.78)
4	8,948 (19.38)
5 (most deprived)	9,557 (20.70)
Missing	679 (1.47)
**Medical Complication**	
Identified	17,173 (37.19)
Not identified	29,003 (62.81)

In the IOL group, the method used to induce labour was ARM alone in 1081 (14.6%), ARM with oxytocin in 2470 (33.4%), prostaglandins with or without ARM in 2639 (35.7%) and prostaglandins and oxytocin with or without ARM in 1211 (16.4%).

### Outcomes of Induction of Labour at 39–41 weeks gestation in women with previous caesarean section

Overall vaginal delivery was achieved in 59.4% of women attempting IOL between 39 and 42 weeks (4,399/7,401), with 37.8% (2,797/7,401) having spontaneous vertex delivery and 21.6% (1,602/7,401) having instrumental delivery (ventouse or forceps). 40.1% (2,969/7,401) of women who underwent IOL were ultimately delivered by caesarean. The mode of delivery was unknown in 34/7,401 cases (0.5%).

In this IOL cohort 49 women had a uterine rupture (0.66%) and in 5 of these cases the baby died. These deaths represent 10.2% of uterine ruptures and 0.07% of all women with IOL and a previous caesarean scar. The rate of uterine rupture was higher when IOL was performed with prostaglandins than without (34/3,862 [0.88%] versus 17/3,539 [0.48%]; p 0.048). 1,319 (17.82%) women undergoing IOL had a postpartum hemorrhage.

916 babies (12.38%) were admitted to a neonatal unit or special care unit, and the extended perinatal mortality rate was 3.4 per 1,000 deliveries (25/7,401).

### Mode of delivery associated with IOL at 39, 40 and 41 weeks gestations compared to expectant management in women with previous caesarean section

Table 2 shows the mode of delivery associated with IOL at 39, 40 and 41 weeks, when compared to expectant management. At all gestations, IOL was associated with lower odds of caesarean delivery on multivariable analysis (Adjusted Odds Ratio [AOR] [95% CI] of caesarean delivery after IOL at 39 weeks of 0.81 [0.71–0.91], after IOL at 40 weeks of 0.72 [0.66–0.79] and after IOL at 41 weeks of 0.70 [0.62–0.79] compared to expectant management) and greater odds of spontaneous vertex delivery (AOR [95%CI] of spontaneous vertex delivery after IOL at 39 weeks compared of 1.14 [1.01–1.29], after IOL at 40 weeks of 1.32 [1.21–1.45] and after IOL at 41 weeks of 1.42 [1.25–1.61] compared to expectant management).

**Table 2:** Mode of delivery associated with induction of labour at 39, 40 and 41 weeks compared to expectant management.

				Univariate Analysis		Multivariable Analysis	
		Expectant Management (%)	IOL (%)	IOL OR (95% CI)	p	IOL AOR (95% CI)	p
	**39w**	13,322/29,064 (45.84)	480/1,268 (37.85)	0.72 (0.64–0.81)	<0.001	0.81 (0.71–0.91)	<0.001
Caesarean Section	**40w**	5,942/12,375 (48.02)	995/2,745 (36.25)	0.62 (0.57–0.67)	<0.001	0.72 (0.66–0.79)	<0.001
	**41w**	993/2,023 (49.09)	1,504/3,388 (44.39)	0.83 (0.74–0.92)	<0.001	0.70 (0.62–0.79)	<0.001
	**39w**	10,250/29,064 (35.27)	501/1,268 (39.51)	1.20 (1.07–1.35)	0.002	1.14 (1.01–1.29)	0.035
Spontaneous Vertex	**40w**	4,037/12,375 (32.62)	1,132/2,745 (41.24)	1.45 (1.33–1.58)	<0.001	1.32 (1.21–1.45)	<0.001
Delivery	**41w**	604/2,023 (29.86)	1,166/3,388 (34.42)	1.23 (1.09–1.39)	<0.001	1.42 (1.25–1.61)	<0.001
	**39w**	5,271/29,064 (18.14)	283/1,268 (22.32)	1.30 (1.13–1.49)	<0.001	1.16 (1.01–1.34)	0.042
Operative Vaginal	**40w**	2,312/12,375 (18.68)	606/2,745 (22.08)	1.23 (1.11–1.36)	<0.001	1.08 (0.97–1.20)	0.16
Delivery	**41w**	421/2,023 (20.81)	700/3,388 (20.66)	0.99 (0.87–1.14)	0.90	1.06 (0.92–1.22)	0.45

Mode of delivery (Caesarean Delivery, Spontaneous Vertex Delivery [SVD] and Instrumental delivery [Forceps or Ventouse]) following induction of labour (IOL) at 39, 40 and 41 weeks (w) in women with a previous caesarean when compared to expectant management (delivery beyond gestation of IOL). Multivariable analysis adjusts for age, period of delivery, deprivation category, presence of medical complication and birth weight. OR = odds ratio. AOR = adjusted odds ratio. 95% CI = 95% confidence interval. * = P<0.05 comparing IOL to expectant management.

### Neonatal and maternal complications associated with IOL and elective repeat caesarean delivery at 39,40 and 41 weeks gestations compared to expectant management in women with previous caesarean section

Overall, there were small numbers of perinatal deaths. There was no significant reduction in adjusted odds of extended perinatal mortality was seen in association with IOL, when compared to expectant management (Table 3). However, elective repeat caesarean delivery was associated with lower odds of extended perinatal mortality when compared to expectant management, with adjusted odds ratio (95% CI) at 39 weeks of 0.23 (0.07–0.75) and no cases of extended perinatal mortality in the caesarean group at 40 and 41 weeks gestation.

**Table 3:** Neonatal and maternal complications associated with induction of labour and repeat caesarean delivery at 39, 40 and 41 weeks each compared to expectant management.

					Univariable Analysis				Multivariable Analysis			
							Repeat				Repeat	
		Expectant		Repeat	IOL		Caesarean		IOL		Caesarean	
		Management	IOL	Caesarean	OR		OR		AOR		AOR	
		(%)	(%)	(%)	(95% CI)	p	(95% CI)	p	(95% CI)	p	(95% CI)	p
Neonatal outcomes												
Perinatal	39w	74/29,064	6/1,268	3/8,931	1.88	0.162	0.13	<0.001	1.37	0.480	0.23	0.0156
Mortality		(0.25)	(0.47)	(0.03)	(0.82–4.32)		(0.04–0.42)		(0.57–3.26)		(0.07–0.75)	
	40w	23/12,375	11/2,745	0/2,999	2.16	0.036	0.18	0.093	1.55	0.274	−n/a	-
		(0.19)	(0.40)	(0)	(1.05–4.44)		(0.02–1.33)		(0.71–3.40)			
	41w	3/2,023	8/3,388	0/1,446	1.60	0.491	n/a	-	5.80	0.106	−n/a	-
		(0.15)	(0.24)	(0)	(0.42–6.02)				(0.69–49.06)			
Admission	39w	3,063/29,064	182/1,268	1,126/8,931	1.42	<0.001	1.2	<0.001	1.29	0.005	0.98	0.59
NNU		(10.54)	(14.35)	(12.61)	(1.21–1.67)		(1.14–1.32)		(1.08–1.55)		(0.90–1.07)	
	40w	1,152/12,375	388/2,745	475/2,999	1.60	<0.001	1.83	0.63	1.39	<0.001	1.08	0.30
		(9.31)	(14.13)	(15.84)	(1.42–1.81)		(1.63–2.06)		(1.21–1.59)		(0.94–1.23)	
	41w	204/2,023	346/3,388	126/1,446	1.01	0.88	0.85	0.18	1.12	0.25	0.77	0.050
		(10.08)	(10.21)	(8.71)	(0.85–1.22)		(0.67–1.07)		(0.92–1.36)		(0.60–1.00)	
Postpartum	39w	4,923/29,064	215/1,268	2,183/8,931	1.00	0.99	1.59	<0.001	1.10	0.27	0.92	0.017
Hemorrhage		(16.94)	(16.96)	(16.96)	(0.86–1.16)		(1.50–1.68)		(0.93–1.30)		(0.86–0.99)	
	40w	2,312/12,375	377/2,745	549/2,999	0.69	<0.001	0.98	0.63	0.96	0.49	0.75	<0.001
		(18.68)	(13.73)	(18.31)	(0.62–0.78)		(0.88–1.08)		(0.84–1.09)		(0.67–0.85)	
	41w	293/2,023	727/3,388	353/1,446	1.61	<0.001	1.91	<0.001	1.38	<0.001	0.81	0.039
		(14.43)	(21.46)	(24.41)	(1.39–1.87)		(1.60–2.27)		(1.17–1.63)		(0.67–0.99)	
Uterine	39w	110/29,064	8/1,268	1/8,931	1.67	0.16	0.03	<0.001	1.79	0.12	0.02	<0.001
Rupture		(0.38)	(0.63)	(0.01)	(0.81–3.43)		(0.00–0.21)		(0.86–3.74)		(0.00–0.13)	
	40w	62/12,375	17/2,745	0/2,999	1.2	0.44	-	-	1.37	0.27		-
		(0.50)	(0.62)	(0)	(0.72–2.12)				(0.78–2.41)			
	41w	11/2,023	24/3,388	1/1,446	1.30	0.47	0.13	0.048	1.26	0.55	0.07	0.011
		(0.54)	(0.71)	(0.07)	(0.64–2.67)		(0.02–0.98)		(0.59–2.68)		(0.01–0.54)	

Neonatal complications (extended perinatal mortality and admission to neonatal special or intensive care unit) and maternal complications (postpartum haemorrhage >1000 ml and uterine rupture) following induction of labour (IOL) or planned caesarean delivery at 39, 40 and 41 weeks (w) in women with a previous caesarean when compared to expectant management (delivery beyond gestation of IOL). Multivariable analysis adjusts for age, period of delivery, deprivation category, presence of medical complication, birth weight and mode of delivery (adjustment for mode of delivery not applied to uterine rupture). OR = odds ratio. AOR = adjusted odds ratio. 95% CI = 95% confidence interval. * = P<0.05 comparing IOL or planned caesarean delivery to expectant management.

IOL at 39 and 40 weeks gestation (but not 41 weeks gestation) was associated with greater odds of neonatal unit admission when compared to expectant management, with adjusted odds (95% CI) of neonatal unit admission of 1.29 (1.08–1.55) at 39 weeks and 1.39 (1.21–1.59) at 40 weeks (table 2). In contrast, elective repeat caesarean delivery at 39 and 40 weeks was not associated with a change in odds of neonatal unit admission, but at 41 weeks was associated with a lower odds of neonatal unit admission (adjusted odds ratio [95%CI] 0.77 [0.60–1.00]).

When compared to expectant management, no significant greater odds of uterine rupture was seen associated with IOL (adjusted odds ratio [95%CI] of uterine rupture after IOL at 39 weeks of 1.79 (0.86–3.74), after IOL at 40 weeks of 1.37 [0.78–2.41] and after IOL at 41 weeks of 1.26 [0.59–2.68]). However, elective repeat caesarean delivery at all gestations was associated with lower odds of uterine rupture compared to expectant management (AOR [95% CI] after elective repeat caesarean delivery at 39 weeks of 0.02 [0.00–0.13], at 40 weeks there were no cases uterine rupture associated with elective repeat caesarean delivery, and after planed caesarean delivery at 41 weeks AOR [95%CI] of 0.07 [0.01–0.54] compared to expectant management).

Compared to expectant management, IOL was associated with greater odds of postpartum hemorrhage at 41 weeks (adjusted odds of 1.38 [1.17–1.63]) but not earlier gestations. Elective repeat caesarean delivery was associated with lower adjusted odds of postpartum hemorrhage at all gestations (AOR [95%] of postpartum hemorrhage after elective repeat caesarean at 39 weeks of 0.92 [0.86–0.99], after elective repeat caesarean at 40 weeks of 0.75 [0.67–0.85] and after elective repeat caesarean at 41 weeks of 0.81 [0.67–0.99] compared to expectant management).

## Discussion

In women with singleton pregnancies and one previous caesarean delivery, IOL between 39 and 41 completed weeks achieved vaginal delivery in nearly 60% of women and was associated with a reduction in repeat caesarean deliveries when compared to expectant management. Compared with expectant management, IOL was associated with higher rates of complications, including postpartum haemorrhage for the mother and neonatal unit admission for the baby. Furthermore, there was a non-significant trend for greater extended perinatal mortality, although the absolute risks were small. In contrast, elective repeat caesarean delivery was associated with lower odds of extended perinatal mortality, uterine rupture, postpartum hemorrhage, and neonatal unit admission compared with expectant management.

Using the comparator of expectant management, induction in women with a caesarean section scar does not influence perinatal mortality, whereas in women without a caesarean section scar, perinatal mortality is reduced [9]. In contrast, in women with a previous caesarean section scar, expediting delivery by elective caesarean section is associated with a 75% reduction in the adjusted odds of perinatal mortality. Taken together, a simple explanation could be that expediting delivery at term lowers perinatal mortality compared with doing nothing – but that in those with a previous caesarean section scar, the adverse effects of the induction process itself negates any lowering of perinatal mortality achieved by early delivery.

The strengths of this study are that it used a large unselected population database and it is the first that we are aware of that has explored the risks and benefits of IOL in women with a previous caesarean scar compared to expectant management. The limitations of the study relate to its retrospective design. It is possible that heterogeneity of practice over the time period has influenced the findings, but we have minimized this by adjusting for the period of birth in our analyses. Errors in coding are another potential source of bias, however quality assurance indicates that fields used in our study have fewer than 2% errors except estimated gestation (error 8%) and induction of labour (error 7%).[21]. ICD codes used to determine medical indication for IOL are liable to greater degrees of error, but these have a tendency for under-recording of conditions. It is probable that a higher proportion of women in the IOL and elective repeat caesarean delivery groups had unrecorded medical conditions than in the expectant management group, as these may indicate delivery. As the presence of medical condition is associated with complications it is likely that estimates of the maternal benefits of IOL and elective repeat caesarean delivery compared to expectant management are conservative.

Although the retrospective study design is associated with limitations, in the absence of large randomised clinical trials, which considered to be unfeasible [4], our study provides an important contribution to the evidence about the pros and cons of IOL in women with a uterine scar. We have attempted to determine the risks and benefits of IOL compared to an expectant (“wait and see”). We have also determined the odds of the alternative approach, elective repeat caesarean delivery, again in comparison with expectant management. This use of an expectant management group, although superficially counterintuitive, is now considered the most clinically relevant comparator group to those undergoing induction of labour [19], as it is more representative of the choice available to woman and their caregivers when considering options for delivery.

We did not find a significant increase in rates of uterine rupture in association with IOL in women with a previous caesarean scar, compared to expectant management. However, uterine rupture is an infrequent event, leading to wide confidence limits so that a 2–3 fold greater odds of uterine rupture in the IOL compared to the expectant group cannot be excluded. In contrast, the odds of uterine rupture were significantly lower in the caesarean section compared to the expectant management group. Observational studies which have compared outcomes in women with a previous caesarean section who have been induced with those of women who have laboured spontaneously have provided conflicting results as to whether IOL is [6], [22]–[27] or isn't [5], [28], [29] associated with uterine rupture. Our data suggests that avoiding IOL and awaiting spontaneous labour does not significantly protect women from uterine rupture, although elective caesarean does.

We found IOL in women with a previous caesarean scar was associated with greater rates of postpartum hemorrhage and neonatal unit admission compared to expectant management, whereas elective repeat caesarean delivery reduced these risks. Additionally, extended perinatal mortality was lower in the elective caesarean section group but not in the IOL group, when each were compared with expectant management. This supports the findings of Crowther et al, and suggests that, when the outcomes for the index pregnancy are considered alone, elective repeat caesarean delivery may be the “safer” option in women with a previous caesarean scar [4]. Nevertheless, IOL was associated with a decrease in repeat caesarean delivery, compared with expectant management. Given that a woman has had two caesarean deliveries is highly likely to be delivered by caesarean in any subsequent pregnancy, and the increasing recognition of the detrimental effects of multiple caesarean delivery on future health and pregnancy outcome (with maternal morbidity highly correlated to number of caesarean deliveries) [3], if a woman with a previous caesarean scar is considering further pregnancies, then IOL around term to maximise the chance of vaginal delivery may be the appropriate strategy. In contrast, for women with a previous caesarean section scar who consider their second pregnancy is likely to be their last, the apparent reduction in perinatal mortality afforded by elective repeat caesarean section may be attractive.

In conclusion, a more liberal policy of IOL may be one way to reduce repeat caesarean delivery, but the risks of complications are significant. Whether these are acceptable in order to decrease caesarean delivery is likely to remain controversial and we believe individualized management is appropriate. Further research, including effects on outcomes of subsequent pregnancies and women's views is warranted. However, we hope that this study will provide relevant and valuable information on which women and their clinicians can base decisions about pregnancy management at term.

## Supporting Information

Table S1 **ICD codes and database fields.** Standard International Classification of Diseases (ICD) 9/10 codes and SMR02/SMR11/SBR/SSBIDS/GROS database fields used.
(DOCX)
