# Supplementary material for: Outcomes of Induction of Labour in Women with Previous Caesarean Delivery: A Retrospective Cohort Study Using a Population Database
Source: PLoS One. 2013 Apr 2;8(4):e60404. doi: 10.1371/journal.pone.0060404 (PMC3615029; doi:10.1371/journal.pone.0060404)
Supplement: Table S1 — ICD codes and database fields. Standard International Classification of Diseases (ICD) 9/10 codes and SMR02/SMR11/SBR/SSBIDS/GROS database fields used. (DOCX) [file pone.0060404.s001.docx]

**Table S1**

| **Exclusion Criteria** |
| --- |
| *Malpresentation* (Breech, Shoulder, Cord or not known [SMR02]; O32 Maternal care for known or suspected malpresentation of fetus [ICD 10]; 652 [0,1,3] Malposition and malpresentation of fetus [ICD 9]; 660.0 [0,1,3] Obstruction caused by malposition of fetus at onset of labour [ICD9]); *Abdominal pregnancy* (O36.7 Maternal care for viable fetus in abdominal pregnancy [ICD 10] or O83.3 Delivery of viable fetus in abdominal pregnancy [ICD 10]); *Placenta Previa* (O44.0 Placenta Previa without haemorrhage [ICD 10], 641.0 [0,1,3] Placenta Previa without haemorrhage [ICD 9], O44.1 Placenta Previa with haemorrhage [ICD 10], 641.1 [0,1,3] Haemorrhage from Placenta Previa [ICD 9]); *Prelabour rupture of membranes* (O75.6 Delayed delivery after spontaneous or unspecified rupture of membrane [ICD10]; 658.2 Delayed delivery after spontaneous or unspecified rupture of membranes [0,1,3][ICD9]) |
| **Medical and obstetric conditions** |
| *Hypertensive and renal disorders* (O10 (all) Pre-existing hypertension complicating pregnancy childbirth and the puerperium [ICD10], 642 (all) [0,1,3] Hypertension complicating pregnancy, childbirth and the puerperium [ICD9], O11 (all) Pre-exisiting hypertensive disorder with superimposed proteinuria [ICD10], O12.1 Gestational proteinuria (without hypertension) [ICD10], O12.2 Gestational proteinuria and oedema (without hypertension) [ICD10], O13 (all) Gestational hypertension without significant proteinuria [ICD10], O14 (all) Gestational hypertension with significant proteinuria [ICD10], O15 (all) Eclampsia [ICD10], O16 (all) Unspecified maternal hypertension [ICD10]), O26.8 Other specified pregnancy-related conditions (pregnancy related: Exhaustion and fatigue, peripheral neuritis or renal disease) [ICD10], 646.2 [0,1,3] Unspecified renal disease in pregnancy, without mention of hypertension [ICD9], 646.4[0,1,3] Peripheral neuritis in pregnancy [ICD9]); *Thromboembolic Disease* (O22.3 Deep phlebothrombosis in pregnancy [ICD10]; 671.3 Deep phlebothrombosis, antepartum [0,1,3] [ICD9]; O22.5 Cerebral venous thrombosis in pregnancy [ICD10]; 671.5 Other phlebitis and thrombosis [0,1,3] Cerebral venous thrombosis Thrombosis of intracranial venous sinus [ICD9], O22.9 Venous complication in pregnancy, unspecified [ICD10]; 671.9 Unspecified venous complication [0,1,3] [ICD9]); *Diabetes Mellitus* (O24 (all) Diabetes Mellitus in pregnancy [ICD10]; 648.0 Diabetes Mellitus [ICD9]; 648.8 Abnormal glucose tolerance [ICD9]); *Liver Disorders* (O26.5 Liver disorders in pregnancy, childbirth and the puerperium [ICD10]; 646.7 Liver disorders in pregnancy [ICD9]); *Pre-existing medical disorder* (O99 Other maternal diseases classifiable elsewhere, but complicating pregnancy childbirth and the puerperium [ICD10], 648 Other current conditions in the mother classifiable elsewhere, but complicating pregnancy, childbirth, or the puerperium [0,1,3] (except 648.0 Diabetes Mellitus and 648.8 Abnormal glucose tolerance) [ICD9], 649.4 Epilepsy complicating pregnancy, childbirth, or the puerperium [0,1,3] [ICD9]); *Antenatal complication* (O28 (all) Abnormal findings on antenatal screening of mother [ICD10]) Suspected Fetal abnormality or fetal compromise (O35 (all) Maternal care for known or suspected fetal abnormality or damage [ICD10], 655 (all) [0,1,3] Known or suspected fetal abnormality affecting management of mother [ICD9], 679 Complications of in utero procedures [ICD9], O36 (all) Maternal care for other known or suspected fetal problems (except O36.4 intrauterine death; O36.6 excessive fetal growth and 36.7 Maternal care for viable fetus in abdominal pregnancy) [ICD10], 656 (all) [0,1,3] Other known or suspected fetal and placental problems affecting management of mother (except 656.4 intrauterine death and 656.6 Excessive fetal growth) [ICD9], O43.1 Malformation of Placenta [ICD10], O40 Polyhydramnios [ICD10], 657 Polyhydramnios [ICD9], O41.0 Oligohydramnios [ICD10], 658.0 Oligohydramnios [ICD9], O41.1 Infection of amniotic sac and membranes [ICD10], 658.4 Infection of amniotic cavity [0,1,3] [ICD9], O41.8 Other specified disorders of amniotic fluid and membranes [ICD10], 658.8 Other specified problems associated with amniotic cavity and membranes [0,1,3] [ICD9], O41.9 Disorder of amniotic fluid and membranes, unspecified [ICD10], 658.8 Other specified problems associated with amniotic cavity and membranes [0,1,3] [ICD9], O41.9 Disorder of amniotic fluid and membranes, unspecified [ICD10], 658.9 Other problems associated with amniotic cavity and membranes Unspecified [0,1,3] [ICD9], O43.0 Placental transfusion syndromes [ICD10], Covered in 656 (656.0 Fetal-maternal haemorrhage) [ICD9], O43.8 Other placental disorders [ICD10], Covered in 656 (656.7 Other |
| **Medical and obstetric conditions (continued)** |
| placental conditions) [ICD9], O43.9 Placental disorder, unspecified [ICD10], Covered in 656 (656.9 Unspecified fetal and placental problem) [ICD9], Covered in O36 (O36.2 Maternal care for hydrops fetalis) [ICD10], 678.0 Fetal haematologic conditions [0,1,3] [ICD9]) O45 (all) Premature separation of the placenta [ICD10], 641.2 Premature separation of placenta [0,1,3][ICD9], O46 (all) Antepartum haemorrhage, not elsewhere classified [ICD10], 641.3 Antepartum haemorrhage associated with coagulation defects [0,1,3] [ICD9], 641.8 Other antepartum haemorrhage [0,1,3] [ICD9] 641.9 Unspecified antepartum haemorrhage [0,1,3][ICD9]); *Poor obstetric history* (Previous stillbirth [SSBID] Previous neonatal death [SSBID]. |
| **Outcomes** |
| *Mode of delivery* (Spontaneous Vertex Delivery, Instrumental delivery [ventouse or forceps], Caesarean [emergency and elective] [SMR02]); *Postpartum Haemorrhage* (O72 Postpartum haemorrhage (all) [ICD10]; 666 Postpartum haemorrhage (all) [0,2,4] [ICD9]); *Obstetric Anal Sphincter Injury in women with vaginal delivery* (O70.2 Third degree perineal laceration during delivery [ICD10], 664.2 Third degree perineal laceration [0,1,4] [ICD9]), *Extended perinatal mortality* (Antepartum Intrauterine death, Intrapartum Intrauterine death, Neonatal death excluding deaths from congenital anomlaies [SSBID]); *Admission for neonatal intensive care or special care* (SMR11); *Uterine rupture* (O71.1 Rupture of uterus during labour [ICD10] 665.1 Rupture of uterus during labour [0,1][ICD9]). |
| **Confounding Factors** |
| *Age at delivery* (<20, 20-24, 25-29, 30-34, 35-39, ≥40 [SMR02]); *Parity* (Para 0 or Para ≥1 [SMR02]); *Year of birth* (1981–1985, 1986–1990, 1991–1995, 1996–2000, 2001–2007 [SMR02]); *Deprivation Quintile* (defined by Carstairs 2001 deprivation quintiles 1-5 by postcode); *Birthweight* (categorised as <2500g; 2500-2999g; 3000-3499g; 3500-3999g; 4000-4999g; ≥4500g [SMR02]); *Method of IOL* (Prostaglandins +/-artificial rupture of membranes (ARM)+/- oxytocin, ARM alone, or ARM + oxytocin [SMR02]). |

SMR02=Scottish Morbidity Record 02 (national maternity database); SMR11=Scottish Morbidity Record 11 (national neonatal databases, now replaced by SBR); SBR=Scottish Birth Record; SSBID=Scottish stillbirth and infant death survey (national perinatal mortality database); GROS=General Register Office for Scotland
